# Purification of Flavonoids from Chinese Bayberry (*Morella rubra* Sieb. et Zucc.) Fruit Extracts and α-Glucosidase Inhibitory Activities of Different Fractionations

**DOI:** 10.3390/molecules21091148

**Published:** 2016-08-31

**Authors:** Shuxia Yan, Xianan Zhang, Xin Wen, Qiang Lv, Changjie Xu, Chongde Sun, Xian Li

**Affiliations:** 1 Zhejiang Provincial Key Laboratory of Horticultural Plant Integrative Biology, Zhejiang University, Hangzhou 310058, China; arillaysx@zju.edu.cn (S.Y.); 11216049@zju.edu.cn (X.Z.); 21416039@zju.edu.cn (X.W.); 7lvqiang_85126@126.com (Q.L.); chjxu@zju.edu.cn (C.X.); adesun2006@zju.edu.cn (C.S.); 2 Laboratory of Fruit Quality Biology, the State Agriculture Ministry Laboratory of Horticultural Plant Growth, Development and Quality Improvement, Zhejiang University, Hangzhou 310058, China

**Keywords:** *Morella rubra* fruit, α-glucosidase inhibitory activity, flavonoid glycosides, purification

## Abstract

Chinese bayberry (*Morella rubra* Sieb. et Zucc.) fruit have a diverse flavonoid composition responsible for the various medicinal activities, including anti-diabetes. In the present study, efficient simultaneous purification of four flavonoid glycosides, i.e., cyanidin-3-*O*-glucoside (**1**), myricetin-3-*O*-rhamnoside (**2**), quercetin-3-*O*-galactoside (**3**), quercetin-3-*O*-rhamnoside (**4**), from Chinese bayberry pulp was established by the combination of solid phase extract (SPE) by C18 Sep-Pak^®^ cartridge column chromatography and semi-preparative HPLC (Prep-HPLC), which was followed by HPLC and LC-MS identification. The purified flavonoid glycosides, as well as different fractions of fruit extracts of six bayberry cultivars, were investigated for α-glucosidase inhibitory activities. The flavonol extracts (50% methanol elution fraction) of six cultivars showed strong α-glucosidase inhibitory activities (IC_50_ = 15.4–69.5 μg/mL), which were higher than that of positive control acarbose (IC_50_ = 383.2 μg/mL). Four purified compounds **1**–**4** exerted α-glucosidase inhibitory activities, with IC_50_ values of 1444.3 μg/mL, 418.8 μg/mL, 556.4 μg/mL, and 491.8 μg/mL, respectively. Such results may provide important evidence for the potential anti-diabetic activity of different cultivars of Chinese bayberry fruit and the possible bioactive compounds involved.

## 1. Introduction

Diabetes is an endocrine disorder characterized by elevated blood glucose levels resulting from the defects in insulin-secretory response or resistance to insulin action [1]. One of the therapeutic methods for reducing postprandial hyperglycemia is to delay the absorption of carbohydrates by inhibiting the α-glucosidase activity [2,3]. So far, acarbose, voglibose, and miglitol are frequently used drugs to control hyperglycemia after meals. However, side effects of these compounds, such as flatulence, stomach ache, and diarrhea, are also apparent [4,5,6]. Natural plant products may provide good alternatives for new anti-diabetic compounds with high safety.

Chinese bayberry (*Morella rubra* Sieb. et Zucc.) is an important subtropical fruit tree in the Myricaceae family and the fruit contains high nutritional and medicinal value [7,8,9]. Many bioactive compounds, such as cyanidin-3-*O*-glucoside (C3G) (**1**), myricetin-3-*O*-rhamnoside (M3R) (**2**), quercetin-3-*O*-galactoside (Q3Gal) (**3**), quercetin-3-*O*-rhamnoside (Q3R) (**4**), and quercetin-3-*O*-glucoside (Q3G) have been identified in bayberry [10]. Previously, our results showed that the C3G-rich bayberry fruit extract from the ‘Biqi’ (BQ) cultivar significantly decreased blood glucose levels in streptozotocin (STZ)-induced diabetic mice [11]. Recently, bayberry fruit extracts from nine cultivars were evaluated for the glucose consumption activities in HepG2 cells. Results showed that bayberry cultivars contained higher contents of C3G and three quercetin glycosides (Q3G, Q3Gal, and Q3R) showed higher glucose consumption activities than those cultivars with lower contents of C3G and quercetin glycosides [12]. However, further characterization and comprehensive investigation of these important bioactive compounds from bayberry were limited by the availability of pure compounds and relative high quantities required by further studies. So far, few efficient separation methods have been established for simultaneous purification of these compounds with high recoveries and high yields.

**Figure 1:**
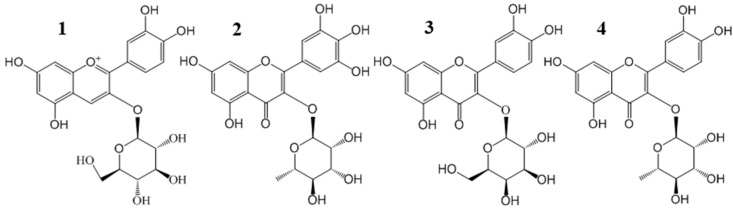
Chemical structures of four main flavonoid glycosides from Chinese bayberry pulp extract: cyanidin-3-*O*-glucoside (C3G) (**1**); myricetin-3-*O*-rhamnoside (M3R) (**2**); quercetin-3-*O*-galactoside (Q3Gal) (**3**); and quercetin-3-*O*-rhamnoside (Q3R) (**4**).

The present study was designed to develop an efficient isolation method to purify C3G and three flavonols (M3R, Q3Gal, and Q3R) (Figure 1) from BQ pulp at the same time, using a combination of solid phase extraction (SPE) by C18 Sep-Pak^®^ cartridges column chromatography and semi-preparative HPLC (Prep-HPLC). α-Glucosidase inhibitory activities and antioxidant activities of the purified flavonoid glycosides, fractions, and extracts of six bayberry cultivars were also evaluated.

## 2. Results and Discussion

### 2.1. Purification of Four Flavonoid Glycosides from BQ Pulp

A simple and efficient method by the combination of column chromatography and Prep-HPLC was established for rapid isolation and purification of four flavonoid glycosides from BQ pulp.

**Figure 2:**
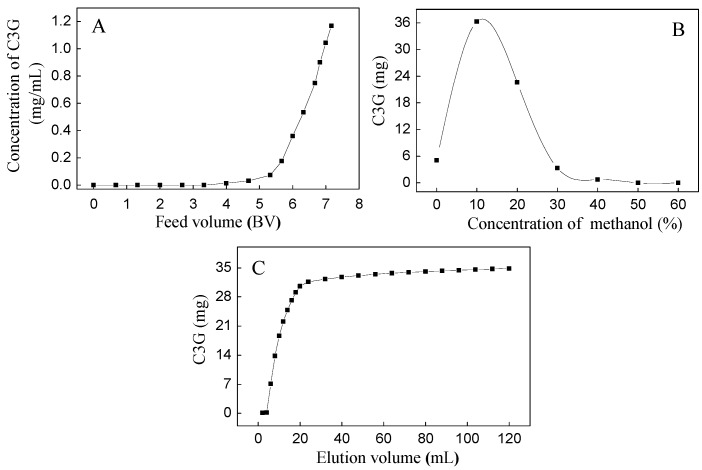
Dynamic leakage curve (**A**); gradient elution curve (**B**); and isocratic desorption curve (**C**) of C3G on a Sep-Pak^®^.

Based on our preliminary experiment, the SPE column showed good isolation between C3G and three flavonols (M3R, Q3Gal, and Q3R). The dynamic adsorption and desorption tests of C3G were first carried out due to their high quantity (Figure 2). The dynamic leakage curves of C3G on the SPE column were obtained based on the volume of effluent and the initial concentration of the sample solution. The initial feed concentration of C3G on SPE column was 1.18 mg/mL. Dynamic adsorption and desorption experiments showed that the breakthrough volume of C3G on SPE column was about 4 bed volume (BV) (Figure 2A), and the majority of C3G absorbed by SPE column was eluted by 10% methanol (with 0.5% formic acid) solution (Figure 2B). Therefore, 10% methanol aqueous solution was used as the eluent in isocratic elution, where complete desorption was done with an elution volume of about 120 mL (10 BV) (Figure 2C). The eluents were collected as 10% fractions, the SPE column was then washed with 50% methanol aqueous solution, and the eluents were collected as a 50% fraction.

**Figure 3:**
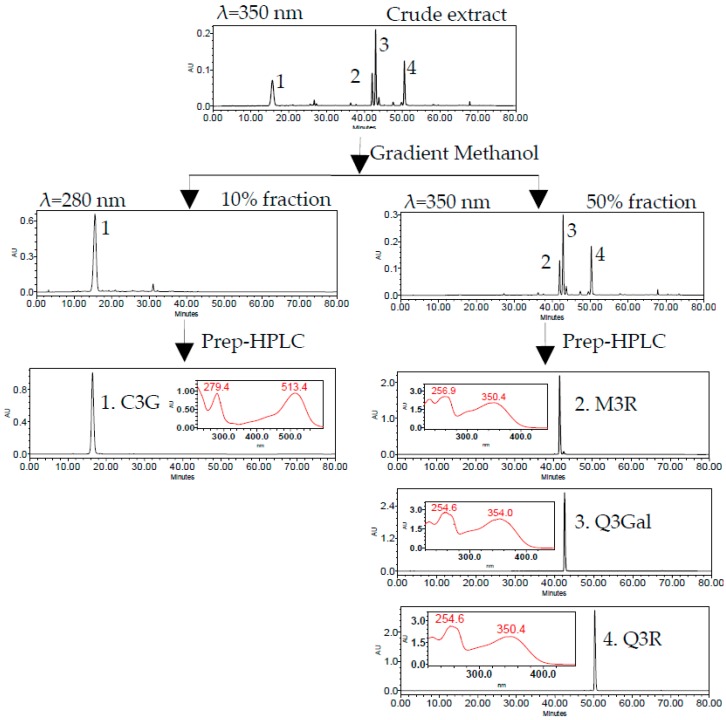
HPLC chromatogram of SPE column and semi-preparative HPLC (Prep-HPLC) purification procedure.

The 10% fraction was rich in C3G (peak 1) and the 50% fraction mainly contained three flavonols, namely, M3R (peak 2), Q3Gal (peak 3), and Q3R (peak 4) (Figure 3). Four flavonoid glycosides were further separated and purified by two different runs of Prep-HPLC. The flow diagram of preparation and purification of four flavonoid glycosides from BQ pulp was shown in Figure 3.

**Figure 4:**
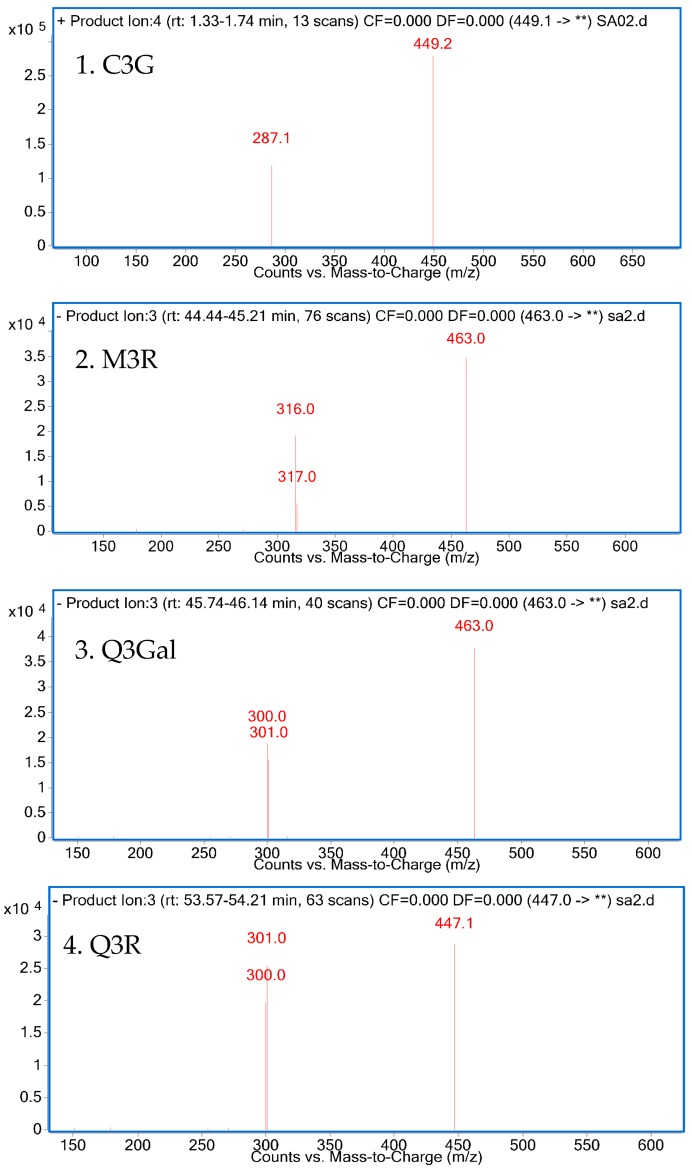
LC-MS^2^ spectrum of four purified products.

The isolated compounds were further identified according to their retention time and UV absorption characteristics by HPLC-DAD analysis (Figure 3). Further structure identification of the purified compounds was confirmed by LC-MS^2^ (Figure 4). For peak 1, the ion at *m*/*z* 449.2 [M + H]^+^ suggested the molecular weight of C3G, and the ion at *m*/*z* 287.1 [M − 162]^+^ represented the glycoside cleavage of one hexoside residue from cyanidin aglycone. For peak 2, the ion at *m*/*z* 463.0 [M − H]^−^ suggested the molecular weight of M3R, and the ion at *m*/*z* 316.0 [M − 146 − 2H]^−^, 317.0 [M − 146 − H]^−^ represented the glycoside cleavage of one hexoside residue from myricetin aglycone. For peak 3 and peak 4, the ion at *m*/*z* 463.0 [M − H]^−^ suggested the molecular weight of Q3Gal, the ion at *m*/*z* 447.1 [M − H]^−^ suggested the molecular weight of Q3R, and the ion at *m*/*z* 300.0 [M − 162 − 2H]^−^, 301.0 [M − 162 − H]^−^ resulted from the cleavage of a hexoside or deoxyhexoside residue from quercetin aglycone. Compared with the previous reports [13], the purified compounds could be confirmed subsequently by our LC-MS^2^ data.

The purities, recoveries, and yields of four flavonoid glycosides in different procedures were summarized in Table 1. The purities of C3G, M3R, Q3Gal, and Q3R in the crude extract of BQ pulp were as low as 0.74%, 0.02%, 0.06%, and 0.03%, respectively. According to the HPLC chromatograms, the crude extract of bayberry fruit was separated into two fractions (Figure 3). After one-step SPE column purification, the purity of C3G, M3R, Q3Gal, and Q3R increased to 54.12%, 2.42%, 7.75%, and 3.84%, respectively, which were about 100-fold enriched than those of the crude extract. The recoveries were all more than 70% (Table 1). Further Prep-HPLC purification resulted in 102.4 mg C3G with 99.17% purity and the recovery rate was 78.18% from 240 mg 10% fraction, and also 4.6 mg M3R with 90.38% purity and the recovery rate was 71.58%, 15.6 mg Q3Gal with 94.57% purity and the recovery rate was 79.32%, 7.6 mg Q3R with 93.61% purity and the recovery rate was 77.20% from 240 mg 50% fraction (Table 1). Such purification procedure was proved as time saving and high efficiency with high yield. The purified compounds can be used for further pharmaceutical research.

### 2.2. Antioxidant Activities Assay

**Figure 5:**
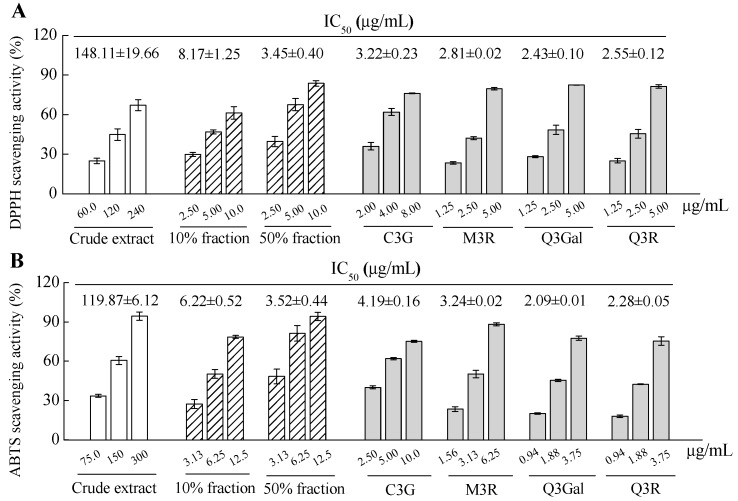
2.2-diphenyl-1-picrylhydrazyl (DPPH) radical scavenging activity (**A**) and 2.2′-azinobis (3-ethylbenzothiazoline-6-sulfonic acid) diammonium salt (ABTS) radical scavenging activity (**B**) of crude extract, fractions, and isolated and four purified compounds from BQ pulp. Data are presented as the mean ± SD (*n* = 3) and IC_50_ was calculated for each sample for both assays.

The antioxidant activity of crude extract, fractions and purified compounds from BQ pulp were evaluated by using both 2.2-diphenyl-1-picrylhydrazyl (DPPH) and 2.2′-azinobis (3-ethylbenzothiazoline -6-sulfonic acid) diammonium salt (ABTS) free radical scavenging assays. Apparent dose-dependent antioxidant activities were observed for all the samples with serial concentrations tested (Figure 5). The IC_50_ value of DPPH scavenging activity of the crude extract was 148.11 μg/mL, while the IC_50_ values for the 10% fraction and 50% fraction were 8.17 μg/mL and 3.45 μg/mL, respectively, for the same assay. For the four purified compounds, higher DPPH scavenging activities were found than those of the crude extract or 10% or 50% fractions, since the IC_50_ values for C3G, M3R, Q3Gal, and Q3R were 3.22, 2.81, 2.43, and 2.55 μg/mL, respectively (Figure 5A). The data from the ABTS scavenging assay showed similar results with the DPPH assay (Figure 5B). Therefore, these four flavonoid glycosides may all contribute to the antioxidant activity of the BQ pulp crude extract.

Antioxidant activity has been proposed by many studies as a key mechanism for the prevention of hyperglycemia associated with diabetes. Recently, our results showed that the extract of BQ fruit could attenuate oxidative stress-induced pancreatic β cell necrosis and protected the insulin secretion ability of the surviving β cell in diabetic mice, which was possibly due to its high antioxidant activity [11,14]. In addition, the glucose consumption activities of the bayberry fruit from different cultivars were significantly correlated to their antioxidant activities [12]. Therefore, the four flavonoid glycosides with high antioxidant activities may play important role to modulate glucose metabolism in vivo.

### 2.3. α-Glucosidase Inhibitory Activity of Different Fractions from BQ Pulp

**Figure 6:**
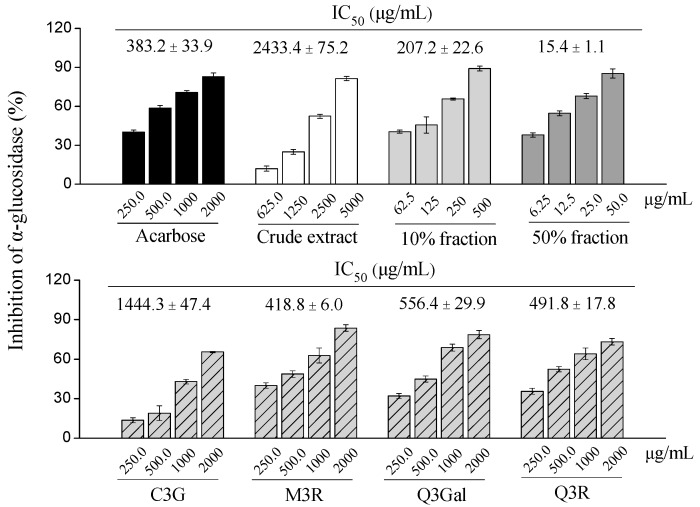
Dose-dependent changes in α-glucosidase inhibition of crude extract, different fractions, and four purified compounds from BQ pulp. Data are presented as mean ± SD (*n* = 3) and acarbose is used as the positive control.

In the present study, different fractions of BQ pulp extract as well as the purified compounds were tested for the α-glucosidase inhibitory activity (Figure 6). Among four purified compounds, the α-glucosidase inhibitory activities were M3R (IC_50_ = 418.8 μg/mL, 902.6 μmol/L) > Q3R (IC_50_ = 491.8 μg/mL, 1097.8 μmol/L) > Q3Gal (IC_50_ = 556.4 μg/mL, 1199.1 μmol/L) > C3G (IC_50_ = 1444.3 μg/mL, 3216.7 μmol/L), which were comparable to the positive control acarbose (IC_50_ = 383.2 μg/mL, 593.6 μmol/L).

Several studies have reported that C3G have beneficial effects for diabetes. The possible action mechanisms include improving insulin sensitivity via modulating the c-Jun N-terminal kinase/fork head box O1 signaling pathway [15], ameliorating hyperglycemia via the reduction of retinol binding protein 4 expression in KK-A^y^ mice [16], or exerting insulin-like activities by PPARγ activation [17]. Q3Gal and Q3R could enhance glucose uptake through stimulating the insulin-independent AMP-activated protein kinase pathway [18,19]. In the present study, our findings suggest that C3G, M3R, Q3Gal, Q3R also had significant α-glucosidase inhibitory activities. Whether they showed similar activity in vivo and whether they have synergistic effects with each other or with other compounds deserve further investigations.

Interestingly, the 50% fraction from BQ pulp extract had the highest α-glucosidase inhibitory activity (IC_50_ = 15.4 μg/mL) than the 10% fraction (IC_50_ = 207.2 μg/mL), the crude extract (IC_50_ = 2433.4 μg/mL) and the four purified compounds, which were also more potent than acarbose (Figure 6).

### 2.4. α-Glucosidase Inhibitory Activity of Six Bayberry Cultivars

**Figure 7:**
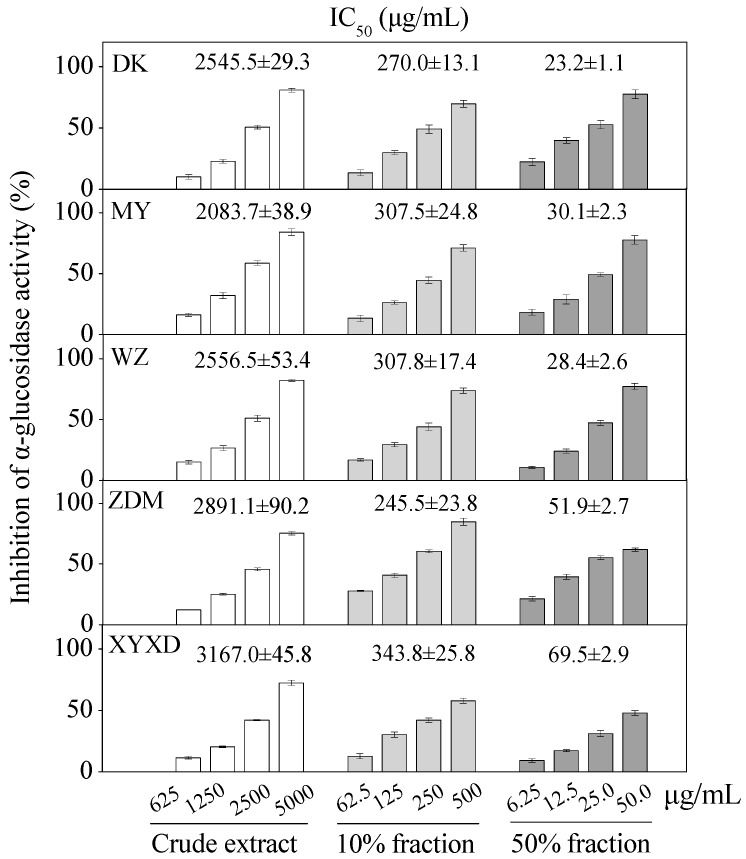
Inhibition of α-glucosidase by crude extract, different fractions from five Chinese bayberry cultivars. IC_50_ values are presented as mean ± SD (*n* = 3).

Apparent dose-dependent α-glucosidase inhibitory activities were observed for six bayberry cultivars with serial concentrations tested (Figure 7). The crude extracts of six bayberry cultivars, i.e., BQ, Dongkui (DK), Muye (MY), Wuzi (WZ), Zaodamei (ZDM), Dayexidi (DYXD), demonstrated α-glucosidase inhibitory activity with IC_50_ values ranging from 2083.7–3167.0 μg/mL and the 10% fractions with IC_50_ values ranging from 245.5–343.8 μg/mL. Furthermore, 50% fractions of six cultivars showed the potent α-glucosidase inhibitory activity with IC_50_ values ranging from 23.2–69.5 μg/mL, all of which were better than that of the positive control acarbose.

The relatively higher α-glucosidase inhibitory activities in the 50% fractions and 10% fractions than those of the purified compounds indicated the involvement of other compounds or the synergistic effects of different bioactive compounds for the α-glucosidase inhibitory effect, which merits further analysis in the future. Additionally, this is the first report about the in vitro α-glucosidase inhibitory activities of different bayberry extracts, and the potent α-glucosidase inhibitory effect of the 50% fraction may provide potential alternatives for the hypoglycemic agents.

## 3. Materials and Methods

### 3.1. Chemicals and Reagents

The authentic C3G, DPPH, dimethyl sulfoxide (DMSO), α-glucosidase (EC 3.2.1.20), methanol, and acetonitrile of chromatographic grade were purchased from Sigma-Aldrich (St. Louis, MO, USA). M3R, Q3Gal, Q3R, acarbose, and 4-nitrophenyl-α-d-glucopyranoside (PNPG) were from J and K Scientific (Shanghai, China). ABTS was bought From Aladdin Inc. (Shanghai, China). Bovine serum albumin (BSA) was purchased from Sangon Biotech (Shanghai, China) Co., Ltd. (Shanghai, China). C18 Sep-Pak^®^ cartridge (12 cc/2 g) columns were obtained from Waters (#WAT036915, Milford, MA, USA). Double-distilled water (ddH_2_O) was used in all experiments and samples for HPLC–DAD and LC-ESI-MS were filtered through a 0.22 μm membrane before injection. All of the other reagents were of analytical grade bought from Sinopharm Chemical Reagent Co., Ltd. (Shanghai, China).

### 3.2. Fruit Materials

Chinese bayberry cultivars BQ, DK, MY, WZ, ZDM, and XYXD fruit were harvested at commercial maturity in June 2014 from Zhejiang province, China. The fruit were selected for uniformity of shape and color, and absence of disease and mechanical damage. The pulp was frozen in liquid nitrogen. After freeze-drying (FM 25EL-85, VirTis, Gardiner, NY, USA), it was ground into a fine powder and stored at −80 °C until extraction and analysis.

### 3.3. HPLC Analysis of Four Flavonoid Glycosides

Flavonoid glycosides were analyzed according to Schieber, Keller, and Carle [20] with some modifications. An HPLC system (2695 pump, 2996 diode array detector, Waters) coupled with an ODS C18 analytical column (4.6 mm × 250 mm) was used for characterizing individual flavonoids compounds. The compounds were detected between 200 and 600 nm. The mobile phase of HPLC consisted of 0.1% (*v*/*v*) formic acid in water (eluent A) and of acetonitrile: 0.1% formic acid (1:1, *v*/*v*) (eluent B). The gradient program was as follows: 0–40 min, 10%–38% of B; 40–60 min, 38%–48% of B; 60–70 min, 48%–100% of B; 70–75 min, 100%–10% of B; 75–80 min, 10% of B. The column was operated at a temperature of 25 °C, the flow rate was 1 mL/min, and the injection volume was 10 μL. Flavonoid glycosides were then quantified based on peak area and comparison with the standard curves. In order to plot the standard curve, independent dilution was made from 2.0 mg/mL with methanol (C3G was with 0.5% formic acid into it) to prepare standard solutions at concentrations of 0.1, 0.2, 0.4, 0.6, 0.8, 1.0, and 2 mg/mL.

### 3.4. Preparation of the Crude Extract

Lyophilized BQ pulp powder (40 g) was extracted with 800 mL of 80% aqueous methanol (with 0.5% formic acid) by sonication for 30 min, while the lyophilized pulp powders (10 g) of the other five (DK, MY, WZ, ZDM, and DYXD) cultivars were extracted with 200 mL of 80% aqueous methanol (with 0.5% formic acid) by sonication for 30 min. The ultrasonic frequency and power were 60 kHz and 30 W, respectively. The crude extract was centrifuged at 10,000 rpm for 10 min at room temperature and the residue was extracted twice as above. All of the supernatants were combined and evaporated by a rotary evaporator under reduced pressure at 37 °C to remove methanol, and then dissolved in ddH_2_O (with 0.5% formic acid).

### 3.5. Dynamic Adsorption and Desorption Tests

In the present study, an SPE column was selected to elute the target compounds. The SPE column was washed by 2 BV of methanol and ddH_2_O successively, and then the crude extract was loaded on the column. The initial feed concentration was 1.81 mg/mL C3G, 0.04 mg/mL M3R, 0.14 mg/mL Q3Gal, and 0.07 mg/mL Q3R. Dynamic adsorption and desorption experiments were first carried out to determine the loading volume and the elution conditions. The column was washed thoroughly by ddH_2_O to remove the impurities. Gradient elution from the SPE column was carried out by increased concentrations of methanol (10%, 20%, 30%, 40%, 50%, and 60% with 0.5% formic acid) successively. Each effluent was collected and analyzed by HPLC. Samples with enriched C3G (10% fraction) and enriched flavonols (50% fraction) were evaporated at 37 °C under vacuum for further Prep-HPLC purification.

### 3.6. Prep-HPLC Purification

The 10% fraction was used to purify individual C3G and the 50% fraction was used to purify the three flavonols by Prep-HPLC (2535 pump, 2998 detector, 2707 auto-sampler, Waters, Milford, MA, USA) with a SunFireTM prep C18 OBDTM (5 μm, 19 mm × 250 mm) column, respectively. The mobile phase solvents were the same as that of HPLC. The C3G gradient program was as follows: 0–10 min, 10% B; 10–30 min, 10%–30% B; 30–50 min, 30%–45% B; 50–60 min, 45%–60% B. The flavonols gradient program was as follows: 0–35 min, 20%–38% B; 35–60 min, 38%–48% B, 60–80 min, and 48%–100% B. The flow-rate was 5.0 mL/min, the injection volume was 300 μL and the concentration was 100 mg/mL. The effluent from the outlet of the column was monitored at 280 nm and 350 nm. The peak fractions were collected according to the chromatogram.

### 3.7. LC-MS Analysis

Mass spectrometric analyses were performed by an Agilent 6460 triple quadrupole mass spectrometer equipped with an ESI source (Agilent Technologies, Santa Clara, CA, USA) that operated in both positive ionization (C3G) and negative ionization (flavonols) mode. The nebulizer pressure was set to 45 psi and the flow rate of drying gas (N_2_) was 5 L/min. The flow rate and the temperature of the sheath gas were 11 L/min and 350 °C, respectively. Chromatographic separations were done as the previous HPLC method. The eluent was split and approximately 0.3 mL/min was introduced into the mass detector. An Agilent Mass Hunter Workstation was used for data acquisition and processing.

### 3.8. Antioxidant Activity

DPPH radical scavenging activity was measured according to Brand-Williams, Cuvelier, and Berset [21] with modifications. The reaction for scavenging DPPH radical was carried out by adding 2 μL sample to 198 μL 25 μg/mL DPPH solution at room temperature. Absorbance at 515 nm before (A_0_) and after (A_1_) the reaction (1 h) was recorded using a micro-plate reader (BioTech, Synergy H1, Winooski, VT, USA). The radical scavenging activity was calculated as:

Scavenging rate (%) = (A_0_ − A_1_)/A_0_ × 100 (1)

The concentration (μg/mL) that inhibited 50% DPPH radicals was calculated as the IC_50_ value.

ABTS assay was carried out using a spectrophotometer as reported [22]. The ABTS radical cation was generated by reacting 7 mmol/L ABTS with 2.45 mmol/L potassium persulfate, and the mixture was allowed to stand in the dark at room temperature for 16 h before use. Before analysis, the ABTS solution was diluted with ethanol to an absorbance of 0.70 ± 0.05 at a wavelength of 734 nm. The absorbance at 734 nm was recorded for 6 min after mixing of 0.1 mL of the tested samples dissolved in ethanol with 3.9 mL of ABTS solution. The radical scavenging activity was calculated as the Equation (1) above. The concentration (μg/mL) that inhibited 50% ABTS radicals was calculated as the IC_50_ value. 

### 3.9. Inhibition of α-Glucosidase Activity

Inhibitory α-glucosidase activity was determined spectrophotometrically in a 96-well microtiter plate based on PNPG as a substrate following the slightly modified method described in [23,24]. α-Glucosidase (20 μL, 0.2 U/mL in 0.01 mol/L potassium phosphate buffer containing 0.2% of BSA) and 0.1 mol/L potassium phosphate buffer (pH 6.8, 112 μL) were mixed with a test sample (8 μL). After pre-incubating at 37 °C for 15 min, 2.5 mmol/L PNPG (50 μL) was added. The reaction was incubated at 37 °C for 15 min and stopped with 0.1 mol/L Na_2_CO_3_ (80 μL). 4-Nitrophenol absorption was measured at 405 nm. Solution with DMSO instead of the sample was used as negative control. Solution without substrate was used as a blank. Acarbose was also assayed as a standard reference. The percent inhibition of α-glucosidase was calculated as follows:

(2) $$\text{Inhibition}\;(\%)=(1-\frac{{\text{OD}}_{\text{test}}\;-\;{\text{OD}}_{\text{blank}}}{{\text{controlOD}}_{\text{test}}\;-\;{\text{controlOD}}_{\text{blank}}})\;\times \;100$$

### 3.10. Statistical Analysis

Experiments were performed in triplicate and data were expressed as the mean ± standard deviation (SD). Data were analyzed using SPSS statistics Version 22 (SPSS Inc., Chicago, IL, USA). All graphical representations were performed using Origin Pro 8.0 software packages (Origin lab Corporation, Northampton, MA, USA).

## 4. Conclusions

An efficient simultaneous purification method combining SPE chromatography with Prep-HPLC was established to isolate four flavonoid glycosides from bayberry fruit in the present study. Purified flavonoid glycosides were identified by HPLC and LC-MS. In addition, four purified compounds were important contributors to the antioxidant and α-glucosidase inhibitory activity of bayberry. Furthermore, the potential high α-glucosidase inhibitory activity of 50% fraction of fruit extracts suggested that the consumption of bayberry fruit after diet might be useful to control the rapid increase of postprandial blood level. Such results may provide an important foundation for further investigation of the pharmaceutical effects of bayberry fruit, as well as the mechanisms acted by different bioactive compounds.

## Figures and Tables

**Table 1:** Purities and recoveries of four flavonoid glycosides in the two-step purification procedure.

Compounds	Purification Step	Purity (%)	Recovery (%)	Yield (mg)
C3G	① Crude extract	0.74	/	/
	② SPE column	54.12	90.75	620.4 ^1^
	③ Prep-HPLC	99.17	78.18	102.4 ^2^
M3R	① Crude extract	0.02	/	/
	② SPE column	2.42	72.24	298.5 ^1^
	③ Prep-HPLC	90.38	71.58	4.6 ^3^
Q3Gal	① Crude extract	0.06	/	/
	② SPE column	7.75	77.11	298.5 ^1^
	③ Prep-HPLC	94.57	79.32	15.6 ^3^
Q3R	① Crude extract	0.03	/	/
	② SPE column	3.84	76.42	298.5 ^1^
	③ Prep-HPLC	93.61	77.20	7.6 ^3^

^1^ The amount of sample was obtained from 50 g freeze dried raw material by the SPE column; ^2^ the amount of compound was obtained from 240 mg SPE column sample (10% fraction) by Pre-HPLC; ^3^ the amount of compound was obtained from 240 mg SPE column sample (50% fraction) by Pre-HPLC.
